# Bibliographical Notices

**Published:** 1839-08-10

**Authors:** 


### BIBLIOGRAPHICAL NOTICES.

*A Short Treatise on Typhus Fever.* By George Leith Roupell, M. D., Physician to St. Bartholomew's Hospital, &c. London: 1839. *Reports from the New York Hospital.* By N. Shook, late Resident Physician. (*From the New York Journal of Medicine and Surgery.*)

This work is from a physician at St. Bartholomew's Hospital, and contains some useful instruction for the management of typhus fever. At the same time, it proves that the author is but indifferently acquainted with the present state of our knowledge upon this subject.

He states, what every one who has studied the subject is fully aware of, that typhus is an exanthematous disease, closely allied to the fevers which are commonly called eruptive, such as measles and small-pox. But the eruption in typhus is so rarely wanting, that the disease is very often designated as petechial fever, from the peculiar measle-like rash, differing from true petechiæ, but more nearly resembling them than any other eruption. Dr. Roupell fancied that this was an original discovery, until he chanced to discover that Dr. Hildenbrand, of Vienna, had arrived at the same conclusion. The lateness of the discovery arose from the great rarity of the work of Dr. Hildenbrand in England; not a single copy in the original could be found in any of the public libraries at London, and the author quotes from a French translation. This is the more singular, as an edition of the work in English was published in this country about ten years ago; it was translated by Dr. Gross, of Cincinnati.

The author afterwards expresses his surprise that Dr. Louis should have laid so much stress upon the lesion of the intestinal follicles in typhus, and disagrees with him as to the frequent occurrence of the lesion; although he regards the diseases described by Dr. Louis as identical with the fever which is almost endemic in Great Britain, and frequently assumes an epidemic form. This is clearly wrong; there is no doubt that typhoid fever exists in Great Britain, just as it does in this country, and that it presents precisely the same anatomical characters as the typhoid form of Paris,—but, on the other hand, the true typhus is without these lesions, which occur only in typhoid fever. It is true that during an epidemic of typhus, the cases of typhoid fever which occur, may assume somewhat of the characteristic appearance of the prevailing disease,—but this is equally true of pneumonia, rheumatism, oı any other acute disease.

The typhus of London in 1838, was very evidently contagious, or, if we please to call it so, infectious, the disease transmitting itself by the exhalations from the patients; hence a great mortality occurred among the physicians and pupils attached to the hospital, as well as the nurses. When a local epidemic occurred at Philadelphia in 1836 and '37, it was very evidently infectious—but few of the nurses attached to the wards in which there were many cases of fever, escaped.

The treatment of an exanthematous disease is, of course, not specific. Still much may be done to relieve the symptoms, and diminish the mortality. The remarks of Dr. Roupell on this subject, show that he is a better practitioner than pathologist. The treatment, in fact, resolves itself into the following general conclusions:

Certain epidemics, and certain periods of an epidemic of typhus fever, require venesection, or at least it will be found beneficial in the majority of cases. To decide as to its propriety, however, there will be much tact necessary on the part of the physician. In general, emetics at the commencement, slightly stimulating diaphoretics, sponging with tepid water, or vinegar and water, are most useful in aiding the natural energies of the system to bring the disease to a close. When there is deficient reaction, particularly towards the close of the acute febrile excitement, wine, or some form of alcohol, is often indispensably necessary. The advantages of this remedy in certain stages of typhus, have been long known, and scarcely ever called in question by sound practitioners. In Philadelphia, the great success of Dr. Parrish in the management of typhus, in 1812 and 1813, arose mainly from his early adoption of this treatment.

The mortality in fevers, it will be seen, is very various. Remittent fevers are strictly under the control of art; and although extremely fatal when of the congestive or malignant form, they may usually be cured by judicious management. Typhus and typhoid fever are not entirely under the control of medicine; hence, if they occur as an epidemic, the mortality must often be considerable, for it depends in a very great degree upon the severity of the disease at a particular season. Thus, we have seen the true typhus in one year prove fatal in a third of the cases—in the next year scarcely destroy one patient in thirty; that is, none died, except a few, who, from some accidental cause, were so enfeebled, that a very slight disorder would prove mortal.

From a report published by Dr. Shook in the New York Journal of Medicine and Surgery, we extract the following interesting remarks on the treatment of remittent fever. The success obtained at the New York Hospital was certainly very good; inasmuch, as of ninety-four patients admitted during the season, only four died—in two of which only, were pathological examinations made. The spleen was extremely enlarged, and softened, the liver enlarged, stomach inflamed, but other viscera free from important disease.

The cases treated at the New York Hospital, were similar to many that are seen at Philadelphia, from the coast of North Carolina. We found that a supporting treatment, with the very early use of quinine, wine, and diaphoretics, was imperatively necessary. The experience of the French army physicians on the northern coast of Africa, accords with our views as to this practice. When, in the congestive, prostrate stage of the fever, blood was abstracted from the patient, the effects were decidedly injurious.

Twenty cases of typhus fever were received into the New York Hospital, during the summer of 1836. These were Irish emigrants; the disease occurred at sea. The remarks of Dr. Shook are in exact accordance with those which we published on the typhus, as observed at Philadelphia, in 1836—'7. The disease ran a determined course,—no specific treatment was discerned, and no decided lesions were observed after death. Glands of Peyer perfectly healthy. Of the twenty cases, six died.

Ten cases of typhoid fever were admitted. Four of these cases terminated fatally. The characteristic disease of the glands of Peyer was present, with perforation into the peritoneum in one of the cases. We are much pleased that the researches which we believe we were the first to make as to the pathology of the fever in the United States, have been fully confirmed by the observations of Dr. Shook. The following remarks are made by him relative to typhoid fever. The memoirs to which he refers, were published in the American Journal of the Medical Sciences, in the years 1835 and '37:

"The symptoms and anatomical lesions all resembled each other, but differed very materially from those of remittent and typhus fever. It is the form of fever which is so common at Paris, and which has been studied with extreme accuracy by Louis and Chomel. Dr. Gerhard, of the Philadelphia Hospital, has given a very able report of the same fever, as witnessed by him during the summer of 1835—'6, showing that the disease differs very much from true typhus, or typhus gravior, ship or jail fever; also, showing from a number of cases examined after death, that the disease is identical with that witnessed at Paris, called typhoid fever, or typhoid affection, or from its anatomical characters, dothinenteritis, (all names belonging to one disease.) The *post mortem* examinations of the four cases that terminated fatally, were carefully noted, as were the symptoms, treatment, and history, previous to death. The anatomical lesions were the same in every case but one, differing, however, very much from those observed in typhus fevers."

The mode of treatment pursued in the cases of remittent fever, is as follows; the extremely small proportion of fatal cases, shows that it was very successful:

"*Remarks on the Treatment of Remittent Fever.*—It appears from the treatment of the preceding cases, that *tonics* were the principal remedies employed. I am aware that tonics have been long given in the stage of convalescence from remittent fever; but the administration of wine, quinine, serpentaria, and nourishing soup, to a patient with a dry, harsh, cracked tongue, dry, hot skin, pulse one hundred and ten, severe aching pains of the limbs, and delirium without any previous depletion, is a practice not so common. It is said that tonics should not be administered when the tongue is dry, and the skin hot and free from moisture, and when there is great pain in the head, with a rapid pulse—in just such cases were wine, porter, and quinine, given with the most gratifying results. The skin became soft and moist; the pulse more calm; the delirium subsided; the tongue immediately began to show that the mucous membranes were acted on, and that an altered state of the secretions was taking place. Tonics not only produced a gradual and permanent influence on the appetite and strength of the patient, but they produced an immediate impression. The improvement was sometimes so rapid as to be very remarkable from one day to the next.

A doubt may arise as to the propriety of using tonics in the early stage of the fever; they were used in all the stages, although at times disadvantages followed their employment; occasionally the quinine would irritate the stomach and increase the fever; if so, (which was rarely the case,) the medicine was discontinued until the severity of the symptoms abated.

The *quinine* was always given in solution, and in small doses; if the excitement was great, the use of it was suspended in the evening.

Immediately after admission into the hospital, the patient's bowels were evacuated by some mild purgative, either rhubarb and magnesia, or castor oil; calomel was seldom employed. The following day, if there was a distinct remission of the fever and no local inflammation to prohibit it, the quinine was given; in most cases, it was not given before the third or fourth day after admission. It was usually continued until the stage of convalescence; together with the quinine was administered an infusion of the roots of the *aristolochia serpentaria*—an ounce to the pint of boiling water. This was given to the patients as a common drink in all stages of the fever. If too bitter and strong, it was diluted with cold water.

*Porter* was given in many cases with decided benefit; but if it produced diarrhœa, it was at once suspended. *Wine-whey* was given to all the patients that were admitted late in the disease with great prostration and exhaustion, even if the tongue was perfectly dry, and the gums covered with sordes; it was surprising to see the rapid improvement in such cases. When given in conjunction with porter, the quantity was about eight ounces daily; but as a temporary prescription to obviate extreme prostration, it was not restricted to this quantity. If care be taken to refrain from wine when there is acute inflammation of some organ, no inconvenience will result from its use.

The *spirit of Mindererus* was given in every case of fever as a sudorific; how far it had the desired effect, independent of the other remedies,

I cannot say, as it was always given in conjunction with them.

The frequent *ablution of the head* and limbs with cold water, was found very agreeable to the patients, and of a good deal of efficacy in equalizing the capillary circulation. Ice water to the head during the height of the fever, was one of the most useful remedies in abating the febrile excitement.

*Anodynes* were given with the most happy effect; nothing appeared to allay nervous irritability and restlessness so well as an anodyne; either ten grains of Dover's powder, or a few drops of the solution of sulphate of morphine. It quieted the patient, abated the delirium, and induced sleep. From the decided benefit that morphine produced in most cases, I should class it amongst the most useful remedies in remittent fever."

---

*Treatise on the Eye, &c.* By WILLIAM CLAY WALLACE, Oculist. Second Edition. New York: 1839. 18mo., pp. 88.

A little volume intended for popular use. It contains a large number of very well executed wood cuts, and is a very lucid exposition of the physiology and comparative anatomy of the eye, as well as of its chief diseases. We think, however, that the author has recommended too highly strychnine, and other dangerous remedies, in the treatment of slight diseases. These are always attended with risk, even under the care of a professional man.

